# Carcass and Meat Characteristics of Cull Heifers from Different Genetic Groups Fed Diets with Different Sources of Nonprotein Nitrogen in Confinement

**DOI:** 10.3390/ani14162304

**Published:** 2024-08-08

**Authors:** Manoel Gustavo Paranhos da Silva, Luís Carlos Vinhas Ítavo, Camila Celeste Brandão Ferreira Ítavo, Marina de Nadai Bonin Gomes, Angelo Herbet Moreira Arcanjo, Jessika Rodrigues de Figueiredo Moura, Brenda Farias da Costa Leite Lopes, Lucimara Modesto Nonato, Rodrigo da Costa Gomes

**Affiliations:** 1 College of Veterinary Medicine and Animal Science, Federal University of Mato Grosso do Sul, Avenida Senador Filinto Muler, 2443, Pioneiros, Campo Grande 79070-900, MS, Brazil; gustavo.paranhos@ufms.br (M.G.P.d.S.); camila.itavo@ufms.br (C.C.B.F.Í.); marina.bonin@ufms.br (M.d.N.B.G.); jessika_rfm@zootecnista.com.br (J.R.d.F.M.); brenda.farias2@hotmail.com (B.F.d.C.L.L.); lucimara.nonato@ufms.br (L.M.N.); 2 Agricultural Research Company of Minas Gerais, EPAMIG Oeste, Rua Afonso Rato, 1301, EPAMIG Oeste, Uberaba 38060-040, MG, Brazil; angelohmarcanjo@gmail.com; 3 Brazilian Agricultural Research Corporation, Embrapa Beef Cattle, Avenida Rádio Maia, 830, Vila Popular, Campo Grande 79106-550, MS, Brazil; rodrigo.gomes@embrapa.br

**Keywords:** collagen, extruded urea, fatty acid profile

## Abstract

**Simple Summary:**

The slaughter of females represents a significant proportion of all cattle slaughtered in Brazil. Despite this, the number of studies on the quality of the carcasses and meat of these animals is still low, which leads to less knowledge about the quality and commercial value of the meat. The aim of this study was to evaluate the carcass and meat quality of heifers from different genetic groups fed diets containing different sources of nonprotein nitrogen. The main differences found were in relation to the genetic group, with effects on carcass weight, fat content in the carcass and meat, and also on the fatty acid profile of the meat.

**Abstract:**

The aim of this study was to evaluate the effect of genetic groups and diets with different sources of nonprotein nitrogen (NPN) on the carcass and meat characteristics of beef heifers. The meat from 40 heifers (20 ½ Angus ½ Nellore (A × N) and 20 ½ Charolais ½ Nellore (L × N)), finished in feedlots, was used. The heifers were fed diets containing different sources of NPN—(1) a diet with livestock urea and protected urea (LPU) and (2) a diet with extruded urea (EU)—in a completely randomized design with a 2 × 2 factorial arrangement. Carcass, composition and meat quality evaluations were carried out. There were no significant interactions between diet and genetic group for most of the variables evaluated (*p* > 0.05). The A × N heifers had higher hot carcass weights (305.73 vs. 279.80 kg), loin eye areas (80.87 vs. 75.45 cm^2^), subcutaneous fat thicknesses (8.69 vs. 6.35 mm) and lower shear forces (6.98 vs. 7.7 kg) compared to the C × N heifers (*p* < 0.05). The meat from the A × N heifers had higher proportions of saturated fatty acids (49.41 vs. 47.95%), with no effects on the proportions of monounsaturated (47.57%) and polyunsaturated (4.01%) fatty acids. The A × N heifers had better carcass and meat characteristics, while the C × N heifers had meat and fat with better fatty acid profiles.

## 1. Introduction

There are many studies in the literature on the carcass and meat characteristics of beef cattle. However, most of these studies were conducted on male cattle. Even if a reasonable proportion of females were included in the total number of cattle slaughtered in Brazil [1,2,3], this would lead to an undervaluation by slaughterhouses. Historically, the meat from cull heifers/cows has been considered be of lower quality than that of male animals.

One of the reasons for the increase in female culling in recent years has been the interest of livestock farmers in inseminating young females, on average 14 months old, in order to shorten the production cycle. Some of the heifers that do undergo insemination fail to become pregnant and are discarded. To make better use of these heifers, they are finished in confinement and can be sold to premium meat markets [4]. They have desirable characteristics, such as good finishing [5], softness and marbling [1].

With the increasing use of crosses between breeds for the production of more efficient animals with better meat quality [4] and the diversified nature of the Brazilian production system, it is essential to evaluate the production performance and carcass and meat characteristics of cull heifers. Such characteristics are determined by aspects intrinsic to the animal [6], food [7] and environment [8].

As with the use of more specialized animals, diet formulation is a critical factor in production. It directly contributes to the growth rate, carcass and meat quality and profitability of the production system [9]. Protein is an essential component of the diet, although it is the most expensive nutrient. Substitutes for this nutrient, such as nonprotein nitrogen (NPN) sources, in finishing diets can reduce feed costs without compromising performance [10,11]. The NNP sources used in ruminant diets have different solubility rates, determining the source or combination of NNP sources that has better synchronization with dietary carbohydrates may increase the microbial protein synthesis and, consequently, improve weight gain [12].

We hypothesized that the carcass and meat characteristics of crossbred cull heifers finished indoors would be unaffected by the genetic group and nonprotein nitrogen sources in the diet. The objective was to evaluate the effect of genetic group and nonprotein nitrogen sources added to the total diet on carcass and meat characteristics of cull heifers (½ Angus ½ Nellore and ½ Charolais ½ Nellore) finished in confinement.

## 2. Materials and Methods

### 2.1. Animals, Treatments and Experimental Design

The experiment was carried out at the Vertente da Pedra Feedlot, located in Água Clara, MS, and at the College of Veterinary Medicine and Animal Science of the Federal University of Mato Grosso do Sul/UFMS. All procedures used in this study were approved by the Ethics Committee for the Use of Animals (ECUA)—UFMS, according to protocol no. 1216/2022. Meat from 40 crossbred cull heifers was used, 20 ½ Angus ½ Nellore (A × N) and 20 ½ Charolais ½ Nellore (C × N), with initial average body weights (BWs) of 374.23 ± 55 kg and ages of 24.0 ± 2 months, kept in the confinement for 102 days. These cull heifers come from the farm’s production system, which discards the heifer after a diagnosis of nonpregnancy in two consecutive insemination protocols.

The foods used in the diets and their compositions are presented in Table 1. Mixed silage (millet silage and Piatã grass planted in intercropped fields), ground corn grain, DDGS (distillery-dried grains with solubles), protected fat (Nutri Gordura^®^, Nutricorp—Araras, SP, Brazil), livestock urea (Reforce-N, Petrobras), protected urea (Prote-N^®^- VitallTech do Brasil- Nutrição Animal, Sarandi, RS, Brazil), extruded urea (Amireia-200 S^®^; Pajoara Ind & Com Ltd.a., Campo Grande, MS, Brazil) and mineral mix (Guabinucleo confinement SPM- Guabi Nutrição e Saúde Animal S. A., Indaiatuba, SP, Brazil).

Amireia is obtained from the extrusion of livestock urea, corn grain and sulfur. The products are ground and subjected to a process involving temperature, pressure and humidity. Protected urea is also produced from livestock urea, but in this case, the urea grain is coated with a polymer. Both products are available on the market as slow-release urea sources.

The diets studied had the same protein and energy contents but different sources of NPN. The first diet (LPU) contained a combination of livestock urea and protected urea, while the second diet (EU) contained only extruded urea as the source of NPN (Table 2). The diets were formulated to meet maintenance requirements and an average daily gain of 1.5 kg, based on the nutritional requirements described in [13]. Metabolizable energy (ME) levels were estimated according to the equations proposed in [13]. The heifers were weighed at the beginning and end of the confinement period after a 16 h fast on solids to determine the average daily gain (ADG), which was calculated by dividing the difference in weight by the confinement period. Dry matter intake (DMI) was determined daily by weighing the amount fed and the leftovers. Feed conversion was estimated by dividing DMI by ADG.

To determine the chemical composition, samples of the ingredients, complete diets and leftovers were analyzed. The samples were dried in a forced-air oven at 55 °C for 72 h and then ground in a knife mill with a 1 mm sieve. Dry matter (DM) was determined in an oven at 105 °C for 24 h (method 930.15 [14]), and the total nitrogen (method 976.05 [14]), ethereal extract (method 920.39 [14]), minerals (method 942.05 [14]) and organic matter were calculated on the basis of the mass loss by combustion. Neutral detergent fiber (NDF) and acid detergent fiber (FDA) were determined by the method described in [15]. The in vitro dry matter digestibility (ivDMD) of the diets was determined using the method described in [16]. Nonfiber carbohydrates (NFCs) were estimated using the equation described in [17], as follows: NFCs = 100 − (%CP + %NDF + %EE + %MM).

The extraction of lipids from the diets was carried out according to the method proposed in [18], using methanol, chloroform and distilled water as reagents. The analysis was carried out on a gas chromatograph with a flame ionization detector (GC/FID) (Thermo, model: Trace GC Ultra). The column used had the following characteristics: 10% cyanopropylphenyl—90% biscyanopropyl polysiloxane stationary phase, 105 m length, 0.25 mm internal diameter, and 0.2 µm film thickness (105 m, the initial temperature was 120 °C for 5 min, with an increase of 5 °C/min until a temperature of 230 °C was reached, which was maintained for 21 min [120 °C (5 min)–230 °C at 5 °C/min–230 °C (21 min)]). Hydrogen (H) was used as the carrier gas at 40 cm 3/s. The injector temperature was 250 °C, with the injection in the split mode with a split ratio of 1:20 and an injection volume of 1.0 µL. Helium gas was used as the carrier gas at a constant flow rate of 1.5 mL/min with a detector temperature of 270 °C. Fatty acids (FAs) were identified by comparing the retention times of the methyl esters in the samples using helium gas as an internal standard. Methyl nonadecanoate (Sigma-Aldrich, São Paulo, SP, Brazil) was used at a concentration of 1 mg/mL in each sample. Fatty acids were quantified by normalizing the area under the methyl ester curve and then estimated using the equation described in [19].

The proportions of the saturated fatty acids (SFAs%), monounsaturated fatty acids (MUFAs%), and polyunsaturated fatty acids (PUFAs%) were calculated in relation to the total amounts of the acids. The sum of the n6 (∑n6) and n3 (∑n3) fatty acids was calculated, and, finally, the ratios of PUFAs/SFAs and n6/n3 were calculated by dividing the amounts of PUFAs by SFAs and n6 by n3 (Table 3)s.

### 2.2. Slaughter and Carcass Evaluations

At the end of the confinement period, the heifers were slaughtered at a commercial abattoir in accordance with the rules established by the Regulations for the Industrial and Sanitary Inspection of Products of Animal Origin—RIISPOA [20]. After slaughter, carcasses were individually identified, sectioned longitudinally, weighed to obtain the hot carcass weight (HCW), and stored at 4 °C for 24 h. The carcass yield (CY) was calculated from the HCW and BW, as follows: CY (%) = (HCW/FBW) × 100.

On the day after slaughter, the left halves of the carcasses were visually evaluated to assess the degree of finishing, fat distribution in the hindquarters, conformation and maturity, according to the methodology described in [21]. Subcutaneous fat distribution was assessed at the heights of the 6th, 9th and 12th ribs, and the following classification scale was used: absent (1.0 ± 0.3), scant (2.0 ± 0.3), median (3.0 ± 0.3), uniform (4.0 ± 0.3) and excessive (5.0 ± 0.3). The conformation of the carcass was assessed visually, and the classification was according to the musculature with variation (+ 0 -); the data were converted into numerical values, as follows: convex—15 to 13; subconvex—12 to 10; rectilinear—9 to 7; subrectilinear—6 to 4; concave—3 to 1.

The carcass pH was then measured between the 12th and 13th ribs, using a portable digital potentiometer with a penetration probe (model HI 99163, Hanna Instruments, Woonsocket, RI, USA), calibrated before analysis with pH 4 and pH 7 standard solutions.

A sample of the longissimus muscle was taken between the 9th and 12th ribs to measure the subcutaneous fat thickness (SFT) and rib eye area (REA). The STF was measured with a digital caliper and classified as follows: <1.0 mm, absent; 1–3 mm, sparse; 3–6 mm, intermediate; 6–10 mm, uniform; and >10.0 mm, excessive. The REA was drawn on tracing paper, and the area (cm^2^) was then estimated using an LI-3100C leaf area meter (Li-Cor Inc., Lincoln, NE, USA). The longissimus muscle sample collected was identified, packed in Styrofoam on ice and then stored at −20 °C until the meat quality analysis.

### 2.3. Meat Composition

The moisture (MO), dry matter (DM), crude protein (CP) and mineral matter (ash) were determined using methods 930.15, 976.05 and 942.05, respectively, as described in [14]. Ethereal extract (EE) was determined using an Ankom XT10 fat extractor (Ankom Technology, NY, USA) in XT4^®^ bags according to method 920.39 [14].

### 2.4. Meat Quality

#### 2.4.1. pH and Color of Meat

The pH of the meat was measured on the thawed steak, and the calibration procedures were the same as those described for assessing the pH of the carcass. The steaks were then kept at room temperature (25 °C) for 30 min, after which the meat color (L*, a* and b*) was measured using a portable spectrophotometer (Meter CR400, Konica^®^ Minolta, Osaka, Japan).

#### 2.4.2. Cooking Losses and Shear Force

Cooking losses and shear force were calculated according to the methodology described by the American Meat Science Association [22]. The steaks were baked in an electric oven (Layr, Crystal model, with upper and lower heating elements, São Paulo, Brazil) until they reached an internal temperature of 71 °C. To monitor the internal temperature of the steaks, rods with temperature sensors (Taylor, model 1478-21, Cincinnati, OH, USA) were inserted into the geometric center of each steak. After reaching the temperature, they were removed from the oven and weighed. Cooking losses were estimated based on the difference in the weights of the steak before and after roasting. After roasting, the steaks were stored for 24 h at 2 °C; then, 7 subsamples of 1.27 cm in diameter were taken from each steak to determine the shear force (SF). The subsamples were taken in the direction of the muscle fiber, using a bench drill. The SF was determined using a texture analyzer (CT3 Warner Bratzler, Brookfield Engineering, Middleborough, MA, USA).

#### 2.4.3. Lipid Oxidation and Myofibrillar Fragmentation Index (MFI)

The lipid oxidation of the meat was assessed by the concentration of malonaldehyde (MDA), mg/kg of meat, according to the methodology described in [23]. The myofibrillar fragmentation index (MFI) was determined according to the method described in [24].

#### 2.4.4. Cholesterol and Collagen Concentration

The total cholesterol concentration was determined by the enzymatic method, according to the methodology described in [25]. The soluble and insoluble collagens were determined from the concentration of hydroxyproline [26]. The collagen concentration in the fresh meat sample (g/100 g) was calculated according to the equations described in [27].

#### 2.4.5. Fatty Acid Profile of Fat and Meat

Lipids were extracted from the meat and fat samples using the technique described in [28]. Fresh samples of 5 g of meat and 1.5 g of fat were used. For the methylation of the fatty acids, the methodology adapted from [29] was applied, and the FA concentrations were calculated using the equations described in [19]. The determination of FAs was carried out following the same procedures used to analyze the diets.

### 2.5. Statistical Analysis

Data were analyzed using a 2 × 2 factorial design with two genetic groups and two diets. All variables were analyzed by analysis of variance using the Proc Glimix procedure in the SAS Studio software, version 9.2 (SAS Institute Inc., Cary, NC, USA) [30], and the means per treatment were compared using the Tukey test at a significance level of 0.05.

## 3. Results

### 3.1. Carcass Evaluation

There was no significant interaction between GG and diet for final weight and carcass characteristics (*p* > 0.05) (Table 4). The A × N heifers had higher final weights (501.42 vs. 483.26 kg), ADGs (1.29 vs. 0.15 kg/day) and DMIs (10.33 vs. 9.92 kg/day) compared to the C × N heifers (*p* < 0.01). A significant interaction was observed for FC, where lower averages were observed in the A × N heifers fed the LPU and EU diets, followed by the C × N heifers fed the LPU diet and, finally, the C × N heifers fed the EU diet (*p* < 0.01).

The HCWs were higher in the A × N heifers, at 305.73 kg, compared to the C × N heifers, at 279.8 kg (*p* < 0.01). The CY and carcass pH were not influenced by the genetic group or diets studied (*p* > 0.05). Measurements of the REA, SFT, and fat distribution were higher in the A × N heifers, at 80.87 cm^2^ and 8.69 mm, compared to the C × N heifers, 75.44 cm^2^ and 6.35 mm (*p* < 0.05), with no effect of diets. There was no significant difference in the physiological maturities (*p* > 0.05).

### 3.2. Meat Composition

There was no effect of GG and diet on the moisture content of the meat (Table 5). A significant interaction was found in the ether extract contents (*p* < 0.05); while the A × N heifers had higher concentrations of EE when they were fed the LPU diet, in the C × N heifers, a greater amount of EE was observed with the EU diet. The concentrations of minerals in the meat was not significant (*p* > 0.05).

### 3.3. Meat Quality

No significant interactions were observed between the genetic group and diet for cholesterol, MFI and lipid oxidation in the meat (*p* > 0.05). Similar trends were found for the levels of total collagen, soluble collagen and insoluble collagen (Table 5). The shear force was 10.5% lower in meat from the A × N heifers (6.97 kg) compared to meat from the C × N heifers (7.70 kg). There was no interactions or significant effects of genetic group and diet on cooking losses (*p* > 0.05).

Significant interaction between the genetic group and diet was found for palmitic (C16:0), palmitoleic (C16:1), heptadecanoic (C17:0), stearic (C18:0), oleic (C18:1n9c), linoleic (C18:2n6c), arachidic (C20:0), y-linolenic (C18:3n6) and behenic (C22:0), with higher concentrations of these acids in the meat from the A × N heifers fed the EU diet (Table 6).

The concentration of capric acid (C10:0) was influenced only by the genetic group, being higher in the C × N heifers, at 0.41 vs. 0.37 (*p* < 0.05). Lauric (C12:0), myristic (C14:0), myristoleic (C14:1), pentadecanoic (C15:0) and α-linolenic (C18:3n3) acids were not influenced by genetic group or diet (*p* > 0.05).

There was a significant interaction between the concentration of arachidonic acid (C20:4n6) and the amount of SFAs in relation to the total amount of acids (*p* < 0.05). The proportion of MUFAs and PUFAs and the amount of n-3 were not influenced by genetic group and diet (*p* > 0.05). There was a significant interaction between genetic group and diet for the n-6 and the n-6/n-3 ratio averages (Table 6).

There was a significant interaction between genetic group and diet for the concentrations of FAs C10:0, C12:0 C14:0, C16:0, C17:0, C18:1n9c, C18:3n6 and C18:3n3, of which greater amounts were observed in the fat of the C × N heifers that received the EU diet (*p* < 0.05) (Table 7).

There was an effect of genetic group on the concentrations of C16:0, C18:3n6, C18:3n3, C14:1 and C16:1, with a higher concentration in the fat of the A × N heifers (*p* < 0.05). There was no effect of genetic group or diet on the concentrations of C15:0 and C18:0 (*p* > 0.05). The amounts of SFAs and MUFAs varied according to genetic group, where fat from the A × N heifers had a higher concentration of SFAs (49.13% vs. 46.78%) and the fat from the C × N heifers had more MUFAs (52.38% vs. 49.28%), with no effect on the PUFA concentration (*p* > 0.05). The sum of n6 was only significant for the NPN content, with a higher average with the EU diet (7.23 vs. 6.84 mg/100 g fat). There was a significant interaction in the sum of n3 (*p* < 0.05), while the concentration of MUFAs and the ratios of PUFAs/SFAs and n6/n3 were not influenced by genetic group or diet (*p* > 0.05).

## 4. Discussion

### 4.1. Carcass Evaluation

The higher final weights of the A × N heifers can be explained by the high genetic potential of the Angus breed, which provides better heterosis when crossing with Nellore [6]. Similar results were also found in [31], which reported higher HCWs, REAs, and SFTs in A × N heifers compared to Nellore or Simmental × Nellore heifers slaughtered at 18 months. Similarly, Ref. [3] recorded higher final weights for A × N cull cows compared to Caracu × Nellore or Nellore cows. On the other hand, Ref. [32] reported a significant difference only in CY and REA for Nellore × Angus cows compared to Angus, Nellore and Hereford cows.

The different solubility rates of the NPN sources may have favored the development of the rumen microbiota and, consequently, better use of nutrients and production of microbial protein, which led to greater cold carcass weights in the A × N heifers. Ref. [12] evaluated the performance of beef steers fed with different sources and combinations of NPN sources, noting that the highest performance was observed with the combination of livestock urea + extruded urea + protected urea, with fast, medium, and slow solubilities, respectively. Recent studies have demonstrated that extruded urea can partially replace true protein sources in ruminant diets [10,11]. According to these works, the advantages of this source of NPN range from a lower risk of intoxication to better synchronization of NPN with other nutrients for microbial protein synthesis and lower dietary cost.

According to [3], slaughter weight has positive correlations with CY, REA and SFT, which may justify the higher REA and SFT values in the A × N heifers. These are characteristics of interest to a slaughterhouse due to their relationship with the yield of commercial cuts and the protection of the carcass during the cooling process [33]. The higher fat content in the A × N heifers also allowed for a better distribution of fat in the carcass. In all treatments, the SFTs were between 6 and 10 mm and classified as uniform finishing, which is much higher than 3 mm minimum required by the slaughterhouse.

In a study that compared the carcass and meat characteristics of A × N cattle of different genders (castrated males, noncastrated males, and females) finished in confinement, with slaughter at 20 months, Ref. [4] reported higher cold carcass weights and REAs in uncastrated males. However, heifers had higher SFTs and marbling, which are factors that influence the sensory attributes of meat [34].

### 4.2. Meat Composition

The lack of differences in the protein, moisture, and ash contents of the meat can be explained by the similarity in ages and finishings, in addition to the animals being of the same sex. As demonstrated in [35] while observing the compositions of meat from cattle of different crosses, the percentages of moisture, crude protein, and ash were little influenced by the genetic group, and only the lipid content showed greater variations. Our results are in line with those described in [35], in which a difference was observed only in the ether extract content. Although the lipid content may vary according to the genetic group, Ref. [2] found no significant difference in the meat compositions of A × N heifers and steers finished in confinement. According to these authors, the lack of significant effect is related to the finishing standard, which was the criterion used for slaughter.

In a study to evaluate the effects of different genetic groups on carcass and meat quality, Ref. [31] found no significant differences in the meat compositions of Nellore, Angus × Nellore and Simmental × Nellore heifers finished in confinement. The average moisture content (72.37%), ether extract (3.12%) and mineral matter (1.31%) are close to those found in this work. There was a difference observed only in the concentration of crude protein, with a higher average in our study (26.06% vs. 20.64%).

### 4.3. Meat Quality

The lower SF observed for the meat from the A × N heifers may be related to better performance. As reported in [36], animals with greater potential for weight gain tend to have lower calpastatin activity, which is directly related to the tenderness of the meat, since calpastatin inhibits the action of calpain, which acts to soften the meat. The SF of meat from the A × N heifers found in this study (6.97 kg) was lower than the 7.91 kg described in [37] while evaluating meat from 148 13-month-old A × N heifers finished in confinement.

The MFI is also related to meat tenderness, with values above 60 indicating high meat tenderness [24]. However, although the MFI results in this study are above this value, the SF data do not confirm the good tenderness of the meat. Lower values for SF could be obtained with maturation for 7 or 14 days, as demonstrated in [3,37]. Another variable linked to meat tenderness is collagen concentration, which is related to genetics, age, animal growth rate, and days of maturation [38]. The absence of significant differences in this variable can be explained by the similarity in the ages and finishes of the heifers at the time of slaughter. Ref. [39] demonstrated through a meta-analysis that there are no differences in the collagen contents of the longissimus muscle among beef breeds, with the same weight and age at slaughter. According to the authors, differences can be found in certain muscles and days of maturation.

Meat color is affected by nutrition, growth rate [40], preslaughter management and, mainly, the animal’s age [41]. As the animal ages, the myoglobin concentration in the meat increases, making it darker [42]. The heifers evaluated in this study were the same age, had the same management during the experiment and preslaughter, and had adequate fat coverage and carcass pH. Although the diets had different sources of NPN, this is not a nutrient that can cause changes in the characteristics of the meat [11].

According to [33], cholesterol and EE levels are associated variables, since EE includes marbling and intracellular fat, where the highest cholesterol levels are concentrated. However, despite the greater amount of EE in the meat of A × N heifers, there was no difference in cholesterol concentration between GG and diet. The cholesterol content found in this study (60.99 mg/100 of meat) was higher than that found in [43] (49.71 mg/100 g meat) in the Longissimus muscle of Nellore × Charolais heifers finished in confinement.

The fatty acid profile of meat changes according to the genetic group and nutrition. Ref. [35] reported that crosses between the Nellore breed and continental breeds produce less C14:0 and C16:0, which was confirmed in this work. The greater amounts of C16:0 and SFAs in the meat of the A × N heifers may also be related to the greater proportion of SFAs in the diet and the greater amount of fat in the carcass. These fatty acids are undesirable because they are related to heart disease and cancer [44].

According to [45], the C14:0 and C16:0 contents arms health when the sum of these two FAs is greater than 35% of the total acids. In the treatments evaluated, the FAs mentioned were below this value, with an average of 31.56 ± 0.81%. Although there was an effect on the amount of SFAs, the amounts of MUFAs and PUFAs were similar among treatments. As described in [35], C18:0 is one of the most abundant acids in beef, but this FA is not related to increased serum cholesterol levels. On the other hand, C18:1n9c and PUFAs are related to the reduction in cholesterol and increase in high-density proteins (HDLs) [46].

The n6 acids were more abundant in the meat of the C × N heifers, which did not differ from the amount found in the meat of the A × N heifers that received the EU diet. Of these acids, the one that had the greatest proportion was C18:2n6. According to [35], this FA is responsible for causing an imbalance in the proportions of n6/n3, where the ideal is a proportion of up to 4.0. The values observed for the proportions of n6/n3 were higher, which may lead to concern, as they are related to heart problems and cancer for the meat consumer. One factor that may have contributed to the high n6/n3 ratio is the small amount of n3 detected in the analysis.

The PUFAs/SFAs ratio was higher in the C × N heifers; however, it was lower than that described in [40] for crosses between Charolais × Caracu (0.21), Angus × Nellore (0.18) and crossbred heifers (0.13). It was also lower than the 0.17 found in [46], which determined the fatty acid profiles of meat from Nellore heifers finished in confinement. The lower PUFAs/SFAs ratio found in this study, compared to the works cited is mainly due to the lower proportion of PUFAs in the total amount of FAs. The amount and proportion of MUFAs in meat can be altered by including seeds or oilseed oil, such as linseed [44], cottonseed, soybean [47], or sunflower [48]. The main FAs impacted by the inclusion of these foods in the diet are n-6 and n-3. Different sources of NPN are not mentioned in the literature as factors capable of altering the fatty acid profile of meat.

The fatty acid profile of the subcutaneous fat was similar to the meat profile for most FAs, with a greater participation of C18:1n9c in both cases. However, differences were observed in the proportion of FAs, with a reduction in the amount of PUFAs and an increase in the proportions of MUFAs. Similar results were found in [49] when analyzing subcutaneous fat from the longissimus muscle of heifers, where the proportions of SFAs and MUFAs were 48.26% and 48.23%, respectively. The proportions of MUFAs (3.42%) and n-3 (0.91%) were higher, while n-6 was higher in our study. The low amount of PUFAs resulted in a low PUFAs/SFAs ratio and, as described for the meat fat, the C18:2n6c acid was responsible for causing an imbalance in the n6/n3 ratio. As reported in [49], fatty acid profiles can vary by type of fat (intramuscular, intermuscular, external and internal) and the amount of fat in the carcass. In the latter case, the increase in fat in the carcass causes an increase in SFAs.

## 5. Conclusions

The A × N heifers had better carcass characteristics and softer meat than the C × N heifers. The meat compositions for heifers from different genetic groups differed only in the fat contents, with higher amounts in the meat from A × N heifers, with no difference among sources of NPN. The C × N heifers had lower concentrations of SFAs in the meat and fat, which provided a better fatty acid profile for this crossbreed. Regardless of the genetic group, the proportion of PUFAs and the PUFAs/SFAs ratios were lower in the subcutaneous fat compared to fat extracted from the meat.

## Figures and Tables

**Table 1:** Chemical composition of the foods used in the experimental diets (g/kg of dry matter).

Item	DM ^1^	OM ^2^	CP ^3^	EE ^4^	NDF ^5^	ADF ^6^	Ash ^7^
Silage ^8^	293.70	964.70	67.18	13.74	755.25	657.76	35.30
Ground corn	872.76	988.83	72.72	22.23	137.48	35.16	11.17
DDGS ^9^	913.25	965.01	415.87	65.47	622.56	314.06	34.99
Protected urea	994.96	999.80	2560.00	58.45	-	-	0.20
Livestock urea	975.09	999.59	2809.10	-	-	-	0.40
Protected fat	963.86	783.77	-	810.85	-	-	216.23
Extruded urea	950.39	995.81	2281.28	26.11	24.73	7.13	4.19
Mineral mix	984.95	118.63	-	-	-	-	881.37

^1^ Dry matter. ^2^ Organic matter. ^3^ Crude protein. ^4^ Ethereal extract. ^5^ Neutral detergent fiber. ^6^ Acid detergent fiber. ^7^ Mineral matter. ^8^ Millet silage and Piatã grass planted and intercropped. ^9^ Distillery dried grains with solubles.

**Table 2:** Formulations and compositions of the experimental diets (g/kg DM).

Item	Diet	
	LPU ^1^	EU ^2^
Silage ^3^	326.2	348.8
DDGS ^4^	71.2	95.5
Ground corn	557.9	519.2
Protected fat	10.0	-
Livestock urea	4.9	-
Protected urea	8.8	-
Extruded urea	-	15.6
Mineral mix ^5^	21.1	20.9
Chemical composition (g/kg DM)		
Dry matter	691.91	678.41
Organic matter	954.63	956.04
Crude protein	151.55	162.20
Ethereal extract	26.64	20.77
Neutral detergent fiber	251.88	275.70
Acid detergent fiber	145.34	163.36
Ash	659.90	596.10
Nonfibrous carbohydrates	691.91	678.41
In vitro dry matter digestibility	954.63	956.04
Metabolizable energy (Mcal/kg DM)	2.72	2.69

^1^ Diet with livestock urea and protected urea. ^2^ Diet with extruded urea. ^3^ Millet silage and Piatã grass planted and intercropped. ^4^ Distillery dried grains with solubles. ^5^ P 27 g/kg; F 27 g/kg; Na 80 g/kg; Mg 25 g/kg; S 32 g/kg; Co 30 mg/kg; Cu 680 mg/kg; I 51 mg/kg; Mn 1.100 mg/kg; Se 9 mg/kg; Zn 2.750 mg/kg; vitamin A 100.000 U.I.; 25-hidroxivitamin D3 20.000 U.I.; vitamin E 600 mg; monensin 1.100 mg/kg; Virginiamycin 730 mg/kg.

**Table 3:** Fatty acid profiles of the experimental diets (mg/100 g lipids in the diet).

Item	Diet	
	LPU ^1^	EU ^2^
C6:0	0.44	0.38
C12:0	0.31	0.29
C13:0	9.20	10.58
C14:0	1.17	-
C16:0	72.22	73.94
C17:0	0.54	0.56
C18:0	22.43	13.88
C18:1n9c	126.92	149.37
C18:2n6c	141.41	175.69
C18:3n6	2.29	2.53
C18:3n3	9.80	12.15
C20:1n9	0.96	1.04
SFAs ^3^ (%)	27.42	22.62
MUFAs ^4^ (%)	32.98	34.15
PUFAs ^5^ (%)	39.59	43.23
∑ n-6	143.70	178.23
∑ n-3	9.80	12.15
PUFAs/SFAs	1.44	1.91
n-6/n-3	14.66	14.67

^1^ Diet containing the combination of livestock urea and protected urea. ^2^ Diet containing extruded urea. ^3^ Saturated fatty acids. ^4^ Monounsaturated fatty acids. ^5^ Polyunsaturated fatty acids.

**Table 4:** Carcass characteristics of beef heifers from different genetic groups finished in confinement.

Item	A × N ^1^		C × N ^2^		SEM ^3^	P > F		
	LPU ^4^	EU ^5^	LPU	EU		GG ^6^	Diet	GG × Diet
Body weight initial (kg)	373.54	372.99	374.23	372.66	2.83	0.95	0.72	0.86
Body weight final (kg)	508.02	494.81	487.52	478.99	3.96	<0.01	<0.01	0.54
Average daily gain (kg/day)	1.30	1.27	1.16	1.14	0.35	<0.01	0.49	0.91
Dry matter intake (kg/day)	10.26	10.39	9.99	9.85	0.10	<0.01	0.94	0.16
Feed conversion ^7^	7.32 d	8.04 c	8.30 b	8.73 a	0.07	<0.01	<0.01	0.04
Hot carcass weight (kg)	302.0	309.45	275.50	284.10	5.91	<0.01	0.18	0.92
Carcass yield (%)	55.65	55.17	55.40	55.30	0.44	0.89	0.52	0.67
pH carcass	5.60	5.65	5.62	5.59	0.04	0.59	0.87	0.40
Rib eye area (cm^2^)	84.05	77.69	76.39	74.50	2.32	0.02	0.08	0.33
Subcutaneous fat thickness (mm)	8.77	8.61	6.64	6.06	0.67	<0.01	0.56	0.74
Fat distribution (score) ^8^	2.05	2.50	1.65	1.80	0.19	<0.01	0.11	0.42
Conformation (score) ^9^	11.30	7.10	8.00	8.10	1.29	0.17	0.11	0.09
Physiological maturity (months)	26.00	25.60	25.60	25.80	1.28	0.94	0.94	0.87

Means followed by different letters differ according to the Tukey test (*p* < 0.05). ^1^ ½ Angus ½ Nellore heifers. ^2^ ½ Charolais ½ Nellore heifers. ^3^ Standard error of the mean. ^4^ Diet containing the combination of livestock urea and protected urea. ^5^ Diet containing extruded urea. ^6^ Genetic group. ^7^ Dry matter intake (kg/day)/average daily gain (kg/day). ^8^ Evaluation scores: absent (1.0 ± 0.3), scant (2.0 ± 0.3), median (3.0 ± 0.3), uniform (4.0 ± 0.3) and excessive (5.0 ± 0.3). ^9^ Evaluation scores: convex—15 to 13; subconvex—12 to 10; rectilinear—9 to 7; subrectilinear—6 to 4; concave—3 to 1.

**Table 5:** Composition and quality of meat from beef heifers from different genetic groups finished in confinement.

Item	A × N ^1^		C × N ^2^		SEM ^3^	P > F		
	LPU ^4^	EU ^5^	LPU	EU		GG ^6^	Diet	GG × Diet
Moisture (%)	72.19	71.96	72.66	72.38	0.26	0.09	0.34	0.93
Crude protein (%)	25.65	26.28	26.48	25.84	0.33	0.58	0.99	0.07
Ethereal extract (%)	3.51 a	2.90 ba	2.21 b	2.73 ba	0.30	<0.01	0.86	0.04
Ash (%)	1.05	1.07	1.07	1.09	1.09	0.44	0.32	0.87
Cholesterol (mg/100 g meat)	57.04	63.30	61.37	62.24	2.67	0.56	0.20	0.33
Myofibrillar fragmentation index	92.30	96.41	97.31	97.95	2.57	0.21	0.36	0.50
Lipid oxidation	2.44	2.95	2.77	2.69	0.43	0.93	0.63	0.51
pH meat	5.62	5.68	5.65	5.60	0.03	0.36	0.99	0.08
Total collagen (g/100 g meat)	0.16	0.16	0.15	0.15	<0.01	0.21	0.98	0.79
Soluble collagen (%)	93.81	94.06	94.65	94.42	0.47	0.19	0.99	0.61
Insoluble collagen (%)	6.18	5.94	5.35	5.58	0.47	0.19	0.99	0.60
Meat color								
L*	37.17	35.62	35.02	36.45	0.78	0.40	0.94	0.06
a*	20.42	18.90	20.30	19.63	0.68	0.66	0.12	0.54
b*	11.15	9.89	10.89	10.62	0.42	0.58	0.08	0.25
Shear force (kg)	7.03	6.92	7.82	7.58	0.22	<0.01	0.44	0.76
Cooking losses (%)	19.78	18.79	18.57	19.81	0.99	0.92	0.89	0.24

Means followed by different letters differ according to the Tukey test (*p* < 0.05). ^1^ ½ Angus ½ Nellore heifers. ^2^ ½ Charolais ½ Nellore heifers. ^3^ Standard error of the mean. ^4^ Diet containing the combination of livestock urea and protected urea. ^5^ Diet containing extruded urea. ^6^ Genetic group.

**Table 6:** Composition of fatty acids (mg/100 g of meat) in meat from beef heifers from different genetic groups fed and finished in confinement.

Item	A × N ^1^		C × N ^2^		SEM ^3^	P > F		
	LPU ^4^	EU ^5^	LPU	EU		GG ^6^	Diet	GG × Diet
C10:0	0.36	0.38	0.40	0.43	0.03	0.03	0.43	0.67
C12:0	0.47	0.46	0.50	0.53	0.04	0.17	0.76	0.61
C14:0	22.28 b	25.12 a	20.87 b	23.74 a	1.3	0.31	0.04	0.99
C14:1	4.14	4.94	4.16	4.61	0.49	0.76	0.21	0.72
C15:0	1.81	2.13	2.16	1.90	0.19	0.73	0.88	0.13
C16:0	187.18 b	220.75 a	183.22 b	183.9 2b	7.50	<0.01	0.03	0.03
C16:1	18.12 b	26.25 a	22.81 ba	24.12 ba	1.69	0.46	<0.01	0.04
C17:0	5.01 b	6.07 a	5.32 ba	5.22 b	0.23	0.24	0.04	0.01
C18:0	101.71 b	120.95 a	101.73 b	100.03 b	4.00	0.01	0.03	0.01
C18:1n9c	277.18 b	342.63 a	293.48 b	290.57 b	13.11	0.18	0.02	0.01
C18:2n6c	15.72 b	21.70 a	20.19 ba	17.60 ba	1.27	0.89	0.19	<0.01
C20:0	0.18 b	0.25 a	0.23 ba	0.21 ba	0.02	0.89	0.11	<0.01
C18:3n6	0.58 b	0.67 a	0.61 ba	0.58 b	0.02	0.07	0.07	<0.01
C18:3n3	1.31	1.22	1.38	1.20	0.12	0.83	0.28	0.77
C22:0	1.62 b	2.49 a	2.23 ba	1.82 b	0.12	0.86	0.21	<0.01
C20:4n6	5.17 b	6.98 a	7.19 a	6.26 b	0.58	0.26	0.44	0.02
SFAs ^7^ (%)	50.18 a	48.63 ba	47.56 b	48.34 ba	0.53	<0.01	0.47	0.03
MUFAs ^8^ (%)	46.42	47.80	48.24	47.80	0.57	0.12	0.41	0.12
PUFAs ^9^ (%)	3.74	3.95	4.35	3.99	0.28	0.21	0.78	0.29
∑ n-6	22.61 b	29.29 a	27.87 a	25.02 ba	1.71	0.77	0.25	<0.01
∑ n-3	1.32	1.22	1.38	1.21	0.12	0.83	0.28	0.77
PUFAs/SFAs	0.07	0.08	0.09	0.08	<0.01	0.08	0.51	0.10
n-6/n-3	19.07 b	25.61 a	20.87 b	20.40 b	1.34	0.19	0.02	<0.01

Means followed by different letters differ according to the Tukey test (*p* < 0.05). ^1^ ½ Angus ½ Nellore heifers. ^2^ ½ Charolais ½ Nellore heifers. ^3^ Standard error of the mean. ^4^ Diet containing the combination of livestock urea and protected urea. ^5^ Diet containing extruded urea. ^6^ Genetic group. ^7^ Saturated fatty acids. ^8^ Monounsaturated fatty acids. ^9^ Polyunsaturated fatty acids.

**Table 7:** Composition of fatty acids (mg/100 g of fat) of fat from beef heifers from different genetic groups fed and finished in confinement.

Item	A × N ^1^		C × N ^2^		SEM ^3^	P > F		
	LPU ^4^	EU ^5^	LPU	EU		GG ^6^	Diet	GG × Diet
C10:0	0.34 b	0.28 b	0.36 ba	0.40 a	0.03	<0.01	0.67	0.04
C12:0	0.49 b	0.39 b	0.51 ba	0.57 a	0.04	<0.01	0.56	0.05
C14:0	24.80 b	21.85 b	22.76 b	28.48 a	1.34	0.09	0.30	<0.01
C14:1	5.38	5.15	5.29	8.24	0.84	0.02	0.30	0.19
C15:0	2.67	2.45	2.48	2.79	0.20	0.72	0.81	0.20
C16:0	180.79 a	172.46 ba	158.25 b	190.82 a	5.85	0.72	0.04	<0.01
C16:1	23.18	22.35	24.34	30.23	2.03	0.03	0.22	0.10
C17:0	5.69b a	5.63 ba	5.15b	6.23 a	0.24	0.92	0.04	0.02
C18:0	97.37	93.12	82.22	96.22	4.31	0.36	0.51	0.10
C18:1n9c	288.06 b	283.44 b	272.28 b	333.69 a	12.05	0.12	0.02	<0.01
C18:2n6c	5.69	6.55	5.78	7.23	0.43	0.38	<0.01	0.50
C18:3n6	0.74 a	0.67 b	0.66 b	0.77 a	0.04	0.96	0.47	0.03
C18:3n3	0.71a	0.58 b	0.62 b	0.73 a	0.05	0.53	0.82	0.02
SFAs ^7^ (%)	49.62	48.64	47.06	46.50	0.64	<0.01	0.22	0.75
MUFAs ^8^ (%)	49.92	51.16	52.47	52.28	0.88	<0.01	0.82	0.64
PUFAs ^9^ (%)	1.14	1.28	1.22	1.24	0.06	0.76	0.20	0.34
∑ n-6	6.44	7.24	6.44	8.01	0.46	0.40	0.01	0.41
∑ n-3	0.72 ba	0.58 b	0.62b a	0.74 a	0.05	0.53	0.82	0.02
PUFAs/SFAs	0.02	0.02	0.02	0.02	<0.01	0.38	0.20	0.30
n-6/n-3	10.2 6	13.62	11.41	11.40	0.99	0.59	0.09	0.09

Means followed by different letters differ according to the Tukey test (*p* < 0.05). ^1^ ½ Angus ½ Nellore heifers. ^2^ ½ Charolais ½ Nellore heifers. ^3^ Standard error of the mean. ^4^ Diet containing the combination of livestock urea and protected urea. ^5^ Diet containing extruded urea. ^6^ Genetic group. ^7^ Saturated fatty acids. ^8^ Monounsaturated fatty acids. ^9^ Polyunsaturated fatty acids.

## Data Availability

Data are contained within the article.

## References

[1] Gallo L., Sturaro E., Bittante G. (2017). Body traits, carcass characteristics and price of cull cows as affected by farm type, breed, age and calving to culling interval. Animal.

[2] Augusto W.F., Bilego U.O., Missio R.L., Guimarães T.P., Miotto F.R.C., Rezende P.L.P., Neiva J.N.M., Restle J. (2019). Animal performance, carcass traits and meat quality of F1 Angus-Nellore steers and heifers slaughtered in feedlot with a similar carcass finishing. Semin. Ciênc. Agrár..

[3] Latta K.I., Ítavo L.C.V., Gomes R.C., Gomes M.N.B., Ferreira J.R., Neves A.P., Araujo T.L.A.C., Feijó G.L.D., Menezes G.R.M. (2024). Carcass characteristics and meat quality of cull cows from different genetic groups. Livest. Sci..

[4] Mueller L.F., Balieiro J.C.C., Ferrinho A.M., Martins T.D.S., Corte R.R.P.S., Amorim T.R., Furlan J.J.M., Baldi F., Pereira A.S.C. (2019). Gender status effect on carcass and meat quality traits of feedlot Angus × Nellore cattle. Anim. Sci. J..

[5] Ferreira I.M., Oliveira K.A., Cidrini I.A., Abreu M.J.I., Sousa L.M., Batista L.H.C., Homem B.G.C., Prados L.F., Siqueira G.R., Resende F.D. (2023). Performance, Intake, Feed Efficiency, and Carcass Characteristics of Young Nellore Heifers under Different Days on Feed in the Feedlot. Animals.

[6] Favero R., Menezes G.R.O., Torres R.A.A., Silva L.O.C., Bonin M.N., Feijó G.L.D., Altrak G., Niwa M.V.G., Kazama R., Mizubuti I.Y. (2019). Crossbreeding applied to systems of beef cattle production to improve performance traits and carcass quality. Animal.

[7] Rezende P.L.D.P., Restle J., Bilego U.O., Fernandes J.J.D.R., Missio R.L., Guimarães T.P. (2019). Carcass characteristics of feedlot-finished Nellore heifers slaughtered at different weights. Acta Sci. Anim. Sci..

[8] Gonzalez-Rivas P.A., Chauhan S.S., Ha M., Fegan N., Dunshea F.R., Warner R.D. (2020). Effects of heat stress on animal physiology, metabolism, and meat quality: A review. Meat Sci..

[9] Greenwood P.L. (2021). Review: An overview of beef production from pasture and feedlot globally, as demand for beef and the need for sustainable practices increase. Animal.

[10] Moraes G.J., Ítavo L.C.V., Ítavo C.C.B., Dias A.M., Niwa M.V.G., Leal E.S., Kozerski N.D., Costa M.C.M., Mata D.G., Inada A.C. (2019). Extruded urea could reduce true protein source in beef cattle diets. J. Anim. Physiol. Anim. Nutr..

[11] Ítavo L.C.V., Ítavo C.C.B.F., Gomes M.N.B., Longhini V.Z., Difante G.S., Dias A.M., Leal E.S., Silva M.G.P., Silva A.H., Silva L.B.P. (2023). Effects of extruded urea levels on the productive performance and carcass and meat characteristics of Nellore cattle. Trop. Anim. Health Prod..

[12] Ítavo L.C.V., Ítavo C.C.B.F., Dias A.M., Franco G.L., Pereira L.C., Leal E.S., Araújo H.S., Souza A.R.D.L. (2016). Combinações de fontes de nitrogênio não proteico em suplementos para novilhos Nelore em pastejo. Rev. Bras. Saúde Prod. Anim..

[13] Valadares Filho S.C., Costa e Silva L.F., Gionbelli M.P., Rotta P.P., Marcondes M.I., Chizzotti M.L., Prados L.F. (2016). Exigências Nutricionais de Zebuínos Puros e Cruzados.

[14] AOAC (2000). Official Methods of Analysis of the Association of Analytical Chemists International.

[15] Mertens D.R. (2002). Gravimetric determination of amylase-treated neutral detergent fiber in feeds with refluxing in beakers or crucibles: Collaborative study. J. AOAC Int..

[16] Ítavo L.C.V., Ítavo C.C.B.F., Valle C.B., Dias A.M., Difante G.S., Morais M.G., Soares C.M., Pereira C.S., Oliveira R.L. (2021). Brachiaria grasses in vitro digestibility with bovine and ovine ruminal liquid as inoculum. Rev. Mex. Cienc. Pecu..

[17] Hall M.B. (2000). Neutral Detergent-Soluble Carbohydrates. Nutritional Relevance and Analysis.

[18] Bligh E.G., Dyer W.J. (1959). A rapid method of total lipid extraction and purification. Can. J. Biochem. Physiol..

[19] Visentainer J.V. (2012). Aspectos analíticos da resposta do detector de ionização em chama para ésteres de ácidos graxos em biodiesel e alimentos. Quim. Nova.

[20] Brasil, 2017. Regulamento de Inspeção Industrial e Sanitária de Produtos de Origem Animal–RIISPOA (Decreto nº 9013, de 29 de março de 2017). Diário Oficial [da] República Federativa do Brasil. https://www.gov.br/agricultura/pt-br/assuntos/inspecao/produtos-animal/arquivos-publicacoes-dipoa/perguntas-e-respostas-decreto-9-013-de-2017-regulamento-de-inspecao-industrial-e-sanitaria-de-produtos-de-origem-animal.

[21] Gomes M.D.N.B., Feijó G.L.D., Duarte M.T., Silva L.G.P., Surita L.M.A., Pereira M.W.F. (2021). Manual de Avaliação de Carcaças Bovinas.

[22] AMSA—American Meat Science Association (2015). Research Guidelines for Cookery, Sensory Evaluation, and Instrumental Tenderness Measurements of Meat.

[23] Vyncke W. (1970). Direct determination of the TBA value in trichloroacetic acid extract of 690 fish as a measure of oxidative rancidity. Fette Seifen Anstrichm..

[24] Culler R.D., Smith G.C., Cross H.R. (1978). Relationship of myofibril fragmentation index to certain chemical, physical and sensory characteristics of bovine longissimus muscle. J. Food Sci..

[25] Saldanha T., Mazalli M.R., Bragagnolo N. (2004). Avaliação comparativa entre dois métodos para determinação do colesterol em carnes e leite. J. Food Sci. Technol..

[26] Hill F. (1966). The solubility of intramuscular collagen in meat from animals of various ages. J. Food Sci..

[27] Cross H.R., Carpenter Z.L., Smith G.C. (1973). Effects of intramuscular collagen and elastin on bovine muscle tenderness. J. Food Sci..

[28] Hara A., Radin N.S. (1978). Lipid extraction of tissues with a low-toxicity solvent. Anal. Biochem..

[29] Maia E.L., Rodriguez-Amaya D.B. (1993). Avaliação de um método simples e econômico para a metilação de ácidos graxos com lipídios de diversas espécies de peixes. Rev. Inst. Adolfo Lutz.

[30] (2024). SAS OnDemand for Academics.

[31] Lage J.F., Paulino P.V.R., Valadares Filho S.C., Souza E.J.O., Duarte M.S., Benedeti P.D.B., Souza N.K.P., Cox R.B. (2012). Influence of genetic type and level of concentrate in the finishing diet on carcass and meat quality traits in beef heifers. Meat Sci..

[32] Mendonça F.S., MacNeil M.D., Nalerio E., Cardoso L.L., Giongo C., Cardoso F.F. (2021). Breed direct, maternal and heterosis effects due to Angus, Caracu, Hereford and Nellore on carcass and meat quality traits of cull cows. Livest. Sci..

[33] Costa E.C.D., Restle J., Vaz F.N., Alves Filho D.C., Bernardes R.A.L.C., Kuss F. (2002). Características da carcaça de novilhos Red Angus superprecoces abatidos com diferentes pesos. R. Bras. Zootec..

[34] Reddy B.V., Sivakumar A.S., Jeong D.W., Woo Y.B., Park S.J., Lee S.Y., Byun J.Y., Kim C.H., Cho S.H., Hwang I. (2015). Beef quality traits of heifer in comparison with steer, bull and cow at various feeding environments. Anim. Sci. J..

[35] Rotta P.P., Prado R.M., Prado I.N., Valero M.V., Visentainer J.V., Silva R.R. (2009). The effects of genetic groups, nutrition, finishing systems and gender of Brazilian cattle on carcass characteristics and beef composition and appearance: A review. Asian-Australas. J. Anim. Sci..

[36] Ramos P.M., Scheffler T.L., Beline M., Bodmer J., Gerrard D.E., Silva S.L. (2023). Challenges and opportunities of using Bos indicus cattle to meet consumers’ demand for quality beef. Meat Sci..

[37] Surita L.M.A., Gomes M.N.B., Dauria B.D., Gomes R.C., Pereira M.W.F., Morais M.G., Duarte M.T., Ítavo L.C.V., Ferraz A.L.J. (2021). Meat quality of cattle subjected to period of aging process: A cross-heifer study. Semin. Ciênc. Agrár..

[38] Purslow P.P. (2018). Contribution of collagen and connective tissue to cooked meat toughness; some paradigms reviewed. Meat Sci..

[39] Blanco M., Jurie C., Micol D., Agabriel J., Picard B., Garcia-Launay F. (2013). Impact of animal and management factors on collagen characteristics in beef: A meta-analysis approach. Animal.

[40] Gómez J.F.M., Antonelo D.S., Beline M., Pavan B., Bambil D.B., Fantinato-Neto P., Saran-Neto A., Leme P.R., Goulart R.S., Gerrard D.E. (2022). Feeding strategies impact animal growth and beef color and tenderness. Meat Sci..

[41] Ponnambalam E.N., Hopkins D.L., Bruce H., Li D., Baldi G., Bekhit A.E.D. (2017). Causes and contributing factors to “dark cutting” meat: Current trends and future directions: A review. Compr. Rev. Food Sci. Food Saf..

[42] Wicks J., Beline M., Gomez J.F.M., Luzardo S., Silva S.L., Gerrard D. (2019). Muscle Energy Metabolism, Growth, and Meat Quality in Beef Cattle. Agriculture.

[43] Macedo L.M.A., Prado I.M., Prado J.M., Rotta P.P., Prado R.M., Souza N.E., Prado I.N. (2008). Chemical composition and fatty acids profile of five carcass cuts of crossbred heifers finished in feedlot. Semin. Ciênc. Agrár..

[44] Wood J.D., Richardson R.I., Nute G.R., Fisher A.V., Campo M.M., Kasapidou E., Sheard P.R., Enser M. (2004). Effects of fatty acids on meat quality: A review. Meat Sci..

[45] Prado I.N., Prado R.M., Rotta P.P., Visentainer J.V., Moletta J.L., Perotto D. (2008). Carcass characteristics and chemical composition of the *Longissimus* muscle of crossbred bulls (*Bos taurus indicus* vs. *Bos taurus taurus*) finished in feedlot. J. Anim. Feed Sci..

[46] Santana E.O., Silva R.R., Simionato J.I., Trindade Júnior G., Lins T.O.D.A., Costa G.D., Mesquita B.M.A., Alba H.D.R., Carvalho G.G.P. (2023). Sex effect on the fatty acid profile and chemical composition of meat from beef cattle fed a whole shelled corn diet. Arch. Anim. Breed..

[47] Valeriano H.H.C., Ítavo L.C.V., Ítavo C.C.B.F., Gomes M.N.B., Dias A.M., Difante G.S., Longhini V.Z., Gurgel A.L.C., Arcanjo A.H.M., Silva M.G.P. (2023). Productive and economic performance of feedlot young Nellore bulls fed whole oilseeds. R. Bras. Zootec..

[48] Ítavo L.C.V., Ítavo C.C.B.F., Dias A.M., Gomes M.N.B., Silva A.G., Leal E.S., Pereira M.W.F., Pereira C.S., Santos G.T. (2021). Lipid rich diet from sunflower seeds can alter the proportion of fatty acids on hybrid Beefalo × Nellore cattle. Trop. Anim. Health Prod..

[49] Sobczuk-Szul M., Mochol M., Nogalski Z., Pogorzelska-Przybyłek P. (2021). Fatty acid profile as affected by fat depot and the sex category of Polish Holstein-Friesian × Limousin fattening cattle fed silage ad libitum. Anim. Sci. J..

